# Case Report: Effect of Targeted Therapy With Carbamazepine in KCNQ2 Neonatal Epilepsy

**DOI:** 10.3389/fneur.2022.942582

**Published:** 2022-07-14

**Authors:** Robertino Dilena, Eleonora Mauri, Alessio Di Fonzo, Cristina Bana, Paola Francesca Ajmone, Claudia Rigamonti, Tamara Catenio, Silvana Gangi, Pasquale Striano, Monica Fumagalli

**Affiliations:** ^1^Neurophysiopathology Unit, Department of Neuroscience and Mental Health, Foundation IRCCS Ca' Granda Ospedale Maggiore Policlinico, Milan, Italy; ^2^Neurology Unit, Department of Neuroscience and Mental Health, Foundation IRCCS Ca' Granda Ospedale Maggiore Policlinico, Dino Ferrari Centre, University of Milan, Milan, Italy; ^3^Child and Adolescent Neuropsychiatic Unit (UONPIA), Foundation IRCCS Ca' Granda Ospedale Maggiore Policlinico, Milan, Italy; ^4^Grioni Center, Danelli Onlus Foundation, Lodi, Italy; ^5^Foundation IRCCS Cà Granda Ospedale Maggiore Policlinico, University of Milan, Neonatology and NICU, Milan, Italy; ^6^Pediatric Neurology and Muscular Diseases Unit, IRCCS ‘G. Gaslini' Institute, Genoa, Italy; ^7^Department of Neurosciences, Rehabilitation, Ophthalmology, Genetics, Maternal and Child Health, University of Genoa, Genoa, Italy

**Keywords:** *KCNQ2*, *SCN2A*, self-limited neonatal epilepsy, carbamazepine (CBZ), developmental and epileptic encephalopathy (DEE), EEG, sodium channel blocker, intronic mutation

## Abstract

We present a family case of neonatal-onset *KCNQ2*-related epilepsy due to a novel intronic mutation. Three members of an Italian family (father and offspring) presented with neonatal-onset asymmetric tonic and clonic seizures with peculiar video-electroencephalography and aEEG features referring to sequential seizures. The father and the first son underwent standard of care treatments in line with current neonatal intensive care unit protocols, with a prolonged hospitalization before reaching full seizure control with carbamazepine. After the experience acquired with her family and the latest advances in the literature, the younger daughter was directly treated with carbamazepine, obtaining rapid seizure control and short hospitalization. They all had normal development. Carbamazepine is rarely administered as a first-line option in neonatal seizures. Recent evidence suggests that neonatal intensive care unit protocols should implement a trial with sodium channel blockers such as carbamazepine as first-option anti-seizure medication and a fast access to genetic testing in neonates with sequential seizures without structural brain injury or acute causes. Moreover, we report and discuss the laboratory studies performed on a novel causative intronic mutation in *KCNQ2* (c.1525+5 G>A in IVS13), since pathogenicity may be difficult to prove for intronic variants.

## Introduction

Seizures are the most frequent neurological sign observed in the neonatal intensive care unit (NICU) (1), and according to etiology, seizures can be classified into structural, metabolic, toxic, infectious, and genetic (2). Early recognition of the specific etiology has a significant impact on therapeutic management of neonatal seizures and neonatal epilepsies.

Genetic neonatal-onset epilepsies (13% of all neonatal seizures) (3) can be further subclassified into structural, metabolic, and functional (4). Functional genetic neonatal-onset epilepsy (8% of all neonatal seizures) (5) may be due to defects in channels, cell signaling, or synaptic transmission (4) with a phenotypic spectrum ranging from self-limited neonatal epilepsy (SLNE, previously referred as benign familial/non-familial neonatal epilepsy) to developmental and epileptic encephalopathy (DEE) (4, 6). Over 80% of SLNE cases are due to mutations in *KCNQ2* (7), and less commonly in *SCN2A* and *KCNQ3* (7).

*KCNQ2, KCNQ3*, and *SCN2A* channelopathies typically present with sequential seizures (2, 4). According to the ILAE consensus sequential seizures are defined by a repetitive succession of asymmetric tonic posturing alternating side, clonic movements, and/or apnea/cyanosis (2, 4). In correspondence to this clinical semiology, video EEG shows a typical sequence (2): electrical decrement (tonic phase) often from one hemisphere (alternating side) is followed by spikes and rhythmic delta-theta activity (clonic phase) and subsequent EEG depression (8). A distinctive ictal pattern has also been observed on aEEG consisting of rapid upper and lower margin rise followed by amplitude depression (8, 9).

This characteristic electroclinical seizure pattern can be encountered both in benign presentations (SLNE) or not (DEE). Interictal EEG with normal background, sleep-wake cycles and physiological graphoelements address a favorable outcome (SLNE) (6), while EEG disorganization with absence of sleep-wake cycles, random attenuation and abundant epileptiform abnormalities, or burst-suppression pattern suggests an unfavorable outcome with disability (DEE) (4). Moreover, DEE is commonly associated with encephalopathy signs, while SLNE is associated with normal neurological examination and positive family history, although it may also have a sporadic presentation (4–6).

Sodium channel blockers and anti-seizure medications such as phenytoin, carbamazepine (CBZ), oxcarbazepine, and lacosamide have been proved effective in neonates with channel-related epilepsy that may be refractory to other therapy (7, 10–14), and around 80% of *KCNQ2-* and *SCN2A*-related epilepsies have a full response to sodium channel blockers. Recognition of sequential seizures addresses diagnosis and treatment early.

Here, we report a family with *KCNQ2*-related epilepsy with a novel intronic mutation and the results of different Neonatal Intensive Care Unit (NICU) therapeutic approaches performed in the different family members due to the evolving knowledge on neonatal-onset epilepsies in the last years and the experiences progressively acquired in the family.

## Case Report

### Sibling I

**Figure 1:**
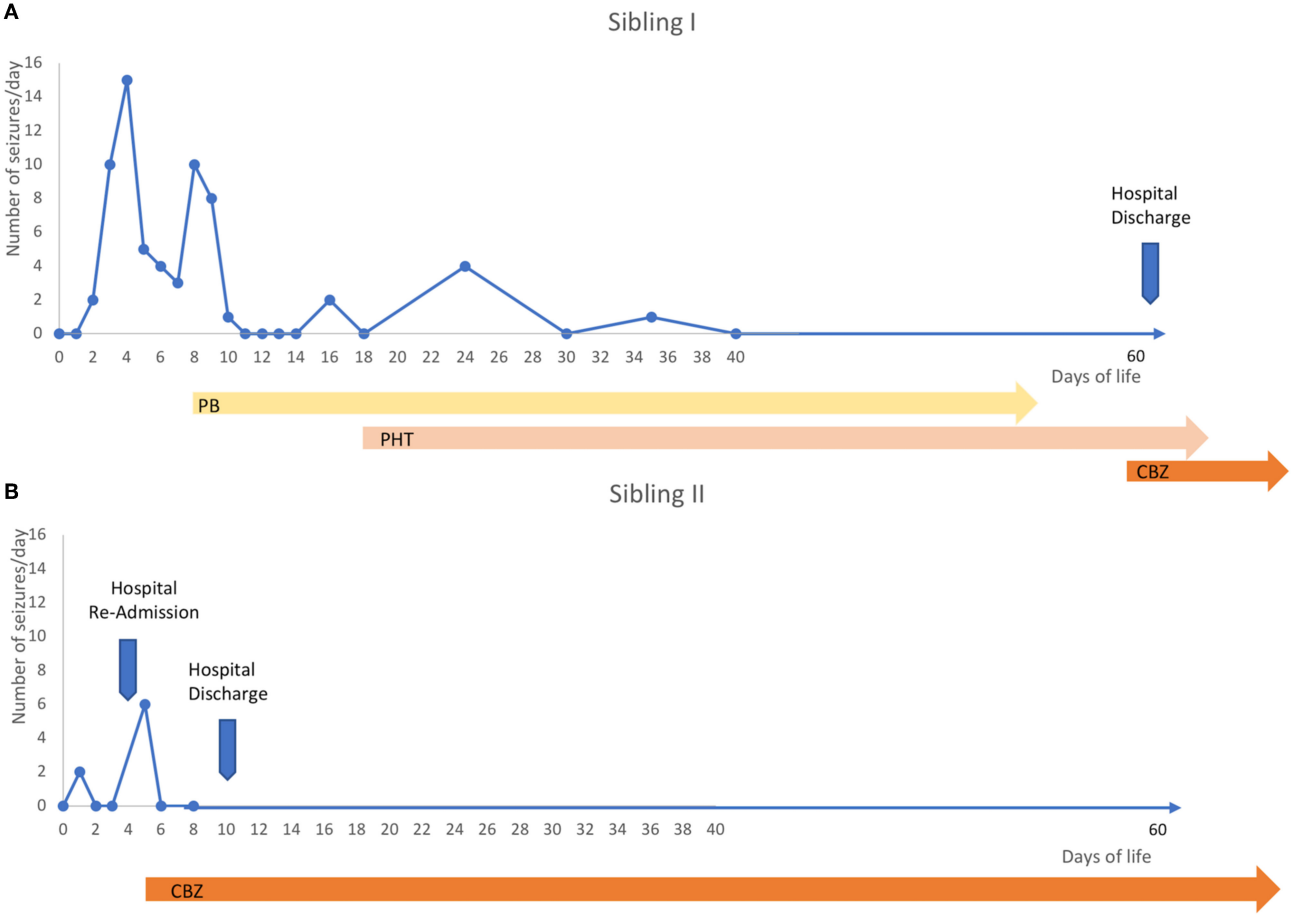
Timeline. **(A)** The first sibling manifested sequential seizures from the second day of life and up to 15 seizures per day in the next days. He was firstly treated with phenobarbital with partial efficacy, so phenytoin was then trialed and it was effective, but seizures relapsed when blood levels were low due to difficulties in maintaining stable drug concentrations in the chronic stage, especially with oral use, so many blood tests were needed to check blood concentrations. Carbamazepine was finally introduced (5 mg/kg twice) at 2 months of age, obtaining stable seizure control. **(B)** One year later, a second sibling was born and had a couple of doubtful paroxysmal events on the second day of life suspicious to be a seizure, so an EEG was performed, but it was normal, so she was discharged. Then, at home, new more clear and frequent events suggestive of seizures occurred, so she was again admitted and the VideoEEG monitoring demonstrated repetitive sequential seizures, so, she was treated with carbamazepine as first choice (5 mg/kg twice) with immediate full seizure control and just five days after she was discharged.

**Figure 2:**
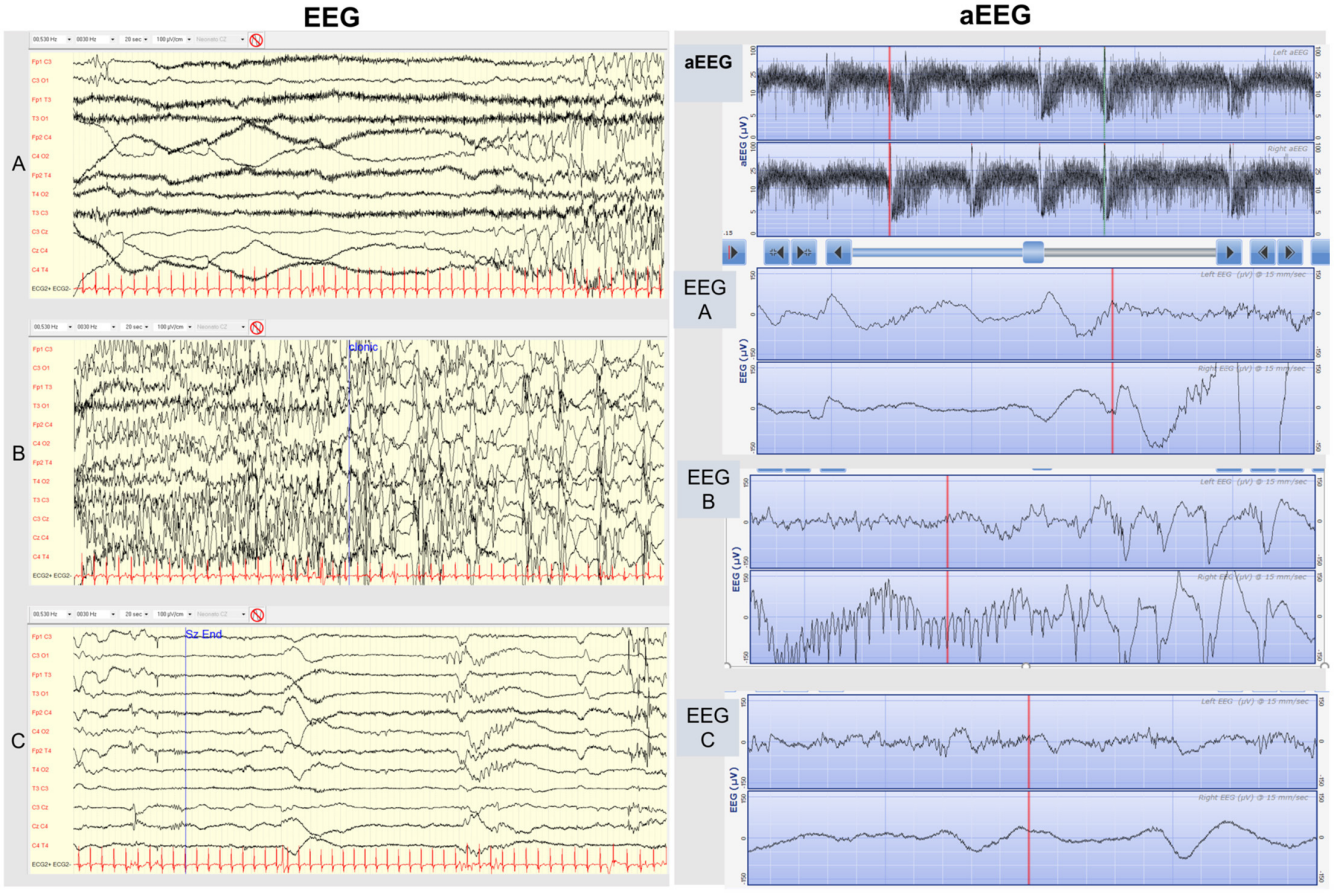
EEG (on the left). Conventional EEG traces of a typical seizure of KCNQ2-related neonatal epilepsy. **(A)** The initial tonic phase is characterized by ictal low-amplitude EEG appearance mixed with muscle artifact. **(B)** The following clonic phase is characterized by rhythmic high-amplitude spike-wave activity. **(C)** Post-ictal low-amplitude depression phase. aEEG (on the right). 2-channel-aEEG (and corresponding EEG raw traces) in KCNQ2-related neonatal epilepsy. Cluster of KCNQ2-related seizures typically characterized by high amplitude peaks enclosed by short troughs. In addition to the first KCNQ2-seizure aEEG pattern description by Vilan et al. we underline the frequent typical appearance in KCNQ2 seizures of a trough preceding the aEEG peak during the initial tonic phase, corresponding in the raw EEG trace to a low-amplitude high-frequency EEG pattern **(EEG A)**, followed by the aEEG peak pattern during the clonic phase, corresponding in the raw EEG to a high-amplitude rhythmic low-frequency spike **(EEG B)**, and finally another trough following the peak during the post-ictal depression phase, corresponding to a low-amplitude EEG pattern **(EEG C)**.

The first son was born at term with an unremarkable perinatal history. From the 2nd day of life, he presented seizures characterized by asymmetric tonic posturing, clonic movements shifting laterality, and apnea up to 15 times a day (Figure 1A). aEEG (Figure 2A) traces showed high-amplitude peaks enclosed by short depressions in correspondence to clinical seizures. Ictal EEG showed electrical decrement for a few seconds, and then rhythmic theta-delta activity with the focus changing in laterality, lasting 60–120 s (Figures 2B,C), whereas background EEG activity was normal. Brain MRI was unremarkable. Phenobarbital was used with partial efficacy. Then, phenytoin was effective during intravenous administration although requiring frequent blood tests to adjust doses and keep therapeutic blood concentrations, but after switching to oral phenytoin administration, seizure relapse occurred and plasma drug levels could not be reached. Finally, oral carbamazepine (5 mg/kg twice a day) was introduced obtaining stable plasma drug levels and was fully effective in seizure control, thus he was finally discharged from neonatal intensive care unit (NICU) (Figure 1A). Carbamazepine was withdrawn after 1.5 years without further relapses.

*KCNQ2* sequencing revealed the novel variant c.1525+5 G>A in the intron or intervening sequence (IVS) 13, which was inherited from the father and considered at that time a variant of unknown significance (VUS). At 2 years of age the general developmental quotient determined by the Griffiths Mental Development Scale was 95 (within normal values, average 100, standard deviation 15). At 6 years of age, psychomotor development was normal, and he was seizure-free.

### Father

The father, in the neonatal age, had epileptic tonic and clonic seizures treated with phenobarbital without full control, and then ACTH was also used. Seizure remission occurred at 8 months of age. At 7 years of age he presented childhood epilepsy with centro-temporal spikes, responsive to carbamazepine, withdrawn at the age of 11 without relapse. Brain MRI was not performed. His neurodevelopment was normal.

### Sibling II

The second sibling was a girl without a remarkable perinatal history. On the 2nd day of life, she manifested a couple of doubtful paroxysmal events suspicious of seizures. An EEG was performed, but it was normal, so she was discharged. At home, new more clear and frequent events suggestive of seizures occurred, so she was again admitted, and the video EEG monitoring demonstrated repetitive sequential seizures, so she was directly treated with oral CBZ 5 mg/kg twice a day with immediate seizure control, allowing discharge from hospital in only 5 days (Figure 1B). Brain MRI did not reveal structural abnormalities or acute brain injuries. She had an epilepsy panel, but only the same intronic mutation was found, and other mutations in other genes involved in epilepsy were excluded. CBZ was withdrawn at 1 year without seizure relapses.

At 10 months of age, her general developmental quotient determined by the Griffiths Mental Development Scale was 100 (within normal values, average 100, standard deviation 15). At 4 years of age, the follow-up of her psychomotor development was normal, and she was seizure free.

### Laboratory

The c.1525+5 G>A IVS13 variant was found in the family in 2015 by *KCNQ2* Sanger sequencing, but at that time, it was considered a VUS. After the second sibling was born, a 72-gene Next Generation Sequencing epilepsy-panel was performed to exclude other mutations, reporting only the same heterozygous variant, segregating within the affected relatives and confirmed by Sanger.

In order to study the effect of the intronic variant found in our family, a transcript expression study on RNA extracted from patient peripheral leukocytes was performed to replicate a study previously applied in another published intronic KCNQ2 mutation (15). A two-round PCR approach was used to overcome the very low expression in blood of *KCNQ2* transcripts, which are brain-specific. However, the transcript sequence analysis on blood-derived cDNA failed to detect splicing aberrations probably because of the limitation of the two-round PCR approach in being able to detect a null transcript or large intron retention as a pathogenetic consequence of the variant.

This variant was absent from clinical and population databases (ClinVar, gnomAD). An *in silico* analysis predicted a high pathogenic probability affecting the normal splicing. On VarSome platform (a search engine, aggregator and impact analysis platform for human genetic variations, that aims at sharing global expertise on human variants) (16), the variant is predicted to be likely pathogenetic, as result of the combination of the strong criterium of pathogenicity PP3 (as the variant is predicting a novel splicing site in gene KCNQ2, for which loss-of-function is a known mechanism of disease) and the moderate criterium of pathogenicity PM2 (as the position is moderately conserved).

## Discussion

We report a family with a novel intronic mutation, c.1525+5 G>A, of the *KCNQ2* gene associated with a self-limited neonatal epilepsy phenotype and a specific response to sodium channel blocker and anti-seizure medications. Since pathogenicity may be difficult to prove for intronic variants, this report may hasten the diagnosis and better treatment in future patients. The report also illustrates how clinical management of functional genetic neonatal epilepsy has greatly changed in the last 8 years thanks to recent scientific advances in clinical, neurophysiological, genetic and pharmacological knowledge (2–14) with a profound impact on patient and family quality of life, although at the moment there is no proof of an improved neurodevelopmental outcome.

Nowadays, the distinction between genetic neonatal-onset epilepsy and provoked seizures could be performed very early (8). The role of neurophysiological evaluation and monitoring by conventional EEG and aEEG has become crucial in clinical practice (17). The recognition of a typical sequential seizure pattern with a prominent tonic onset on video-EEG in a neonate without structural brain damage or acute etiology is an accessible and reliable marker of genetic epilepsy due to *KCNQ2/KCNQ3/SCN2A* channelopathies, allowing for a tailored diagnostic workup and early targeted effective treatment (2–14). Indeed, independently from the neurodevelopmental evolution into SLNE or DEE, neonatal epilepsy associated with channelopathies, *KCNQ2-* or *SCN2A*-related, shows a better response to sodium-channel blockers (2–14) such as phenytoin, oxcarbazepine, and CBZ. In this family, the first son, after 2 months of hospitalization and different anti-seizure medications, obtained full control only after CBZ was started. The second neonate was immediately treated with CBZ with prompt efficacy, markedly shortening hospitalization and preventing complications related to the NICU setting (separation from the mother, infections, and discomfort). The 6-year follow-up of this family also proved normal neurodevelopment after early use of CBZ. Oral CBZ has traditionally been rarely prescribed in neonates (<1% of all neonatal seizures, never as a first-line treatment) (18). There are no specific data on the safety of CBZ use in neonates or infants in comparison with other anti-seizure medications, whereas there are studies on safety of anti-seizure medications during pregnancy (19). Apparently, there is no reason to hypothesize that there could be more side effects with CBZ in neonatal age in comparison with other ages, and studies reporting on the efficacy of CBZ in *KCNQ2* and *SCN2A* neonatal epilepsies did not raise any safety concerns regarding reactions or neurodevelopmental outcomes (2–14). Oral phenytoin is not suitable for chronic use in neonates as plasma drug levels cannot often be achieved by oral administration, as shown in our case and as described by pharmacokinetic studies on phenytoin in neonates (20). The only downside of CBZ is that a parenteral formulation is not available, so in case of status epilepticus with need to rapidly control seizures, initial use of intravenous phenytoin followed by a switch to CBZ oral syrup is suggested (12). An oral alternative to CBZ with a more favorable side effect profile may be represented by oxcarbazepine given its better profile of enzyme induction properties, but not all countries have the availability of oxcarbazepine syrup formulation suitable for infants. A new sodium channel blocker very promising for neonatal seizures is represented by lacosamide, a manageable drug with a good safety profile for which both intravenous and oral syrup formulations have been produced. Lacosamide has been very recently used as an alternative third-option anti-seizure medication for refractory neonatal seizures in some centers (21). Lacosamide has also been demonstrated effective in two neonates with *SCN2A*-related intractable seizures (14). A multicenter randomized controlled study to evaluate efficacy and safety of lacosamide in neonatal seizures described on clinicalTrials.gov (22) is currently recruiting patients. The results of lacosamide trials on neonatal seizures could potentially change the management of neonatal seizures, possibly allowing for its consideration as a first- or second-option targeted treatment for *KCNQ2* or *SCN2A* neonatal epilepsy.

## Conclusions

Early identification of *KCNQ2* variants in patients with epilepsy raises prognostic issues toward optimal management (23). We report a novel intronic mutation of *KCNQ2* associated with familial self-limited neonatal epilepsy. *KCNQ2* and *SCN2A* neonatal epilepsies can be early suspected on the basis of typical clinical, EEG, and aEEG features (sequential seizures with prominent tonic onset semiology) in the absence of acute causes and structural abnormalities. In addition, in inherited cases, family history strongly points to the diagnosis. On the basis of the literature of the last 8 years and experience at our center, we suggest that in suspected *KCNQ2* and *SCN2A* neonatal epilepsies, NICUs should implement fast genetic testing for neonatal epileptic channelopathies and a trial with CBZ. In severe epileptic presentations with status epilepticus, phenytoin could be first started to rapidly obtain seizure control and then in case of positive response, switch to CBZ or oxcarbazepine, as they are more suitable for long-term maintenance. A further promising alternative could be lacosamide given the availability of intravenous and oral formulations and anecdotal evidence showing a good potential for neonatal seizures and channel-related neonatal epilepsies, but larger studies are warranted.

## Data Availability Statement

All data generated or analyzed during this study are included in this article's material files. Further enquiries can be directed to the corresponding author.

## Ethics Statement

The research was conducted ethically following the World Medical Association Declaration of Helsinki and its amendments. Subjects involved and their parents (for minors) have given their written informed consent to publish their case. This study protocol was reviewed and approved by Comitato Etico Milano Area 2 protocol n.0050696.

## Author Contributions

RD conceptualized and wrote the manuscript, performed the neurologic evaluations, collected the clinical history, and performed the neurophysiological assessments. EM conceptualized and wrote the manuscript, collected the clinical, and neurophysiological data. AD performed the genetic analysis and reviewed the manuscript. CB performed the neurophysiological examinations and reviewed the manuscript. CR and TC performed the neuropsychological tests and reviewed the manuscript. SG performed the pediatric evaluation and follow-up and reviewed the manuscript. PS and MF contributed in intellectual content and supervision and reviewed the manuscript. All authors contributed to the article and approved the submitted version.

## Funding

The project received support for APC from the Italian Ministry of Health Ricerca Corrente for 2021.

## Conflict of Interest

RD has received a speaker honorarium from Sobi. SP has received speaker fees and participated on advisory boards for Biomarin, Zogenyx, GW Pharmaceuticals, and has received research funding by ENECTA BV, GW Pharmaceuticals, Kolfarma Srl., Eisa. The remaining authors declare that the research was conducted in the absence of any commercial or financial relationships that could be construed as a potential conflict of interest.

## Publisher's Note

All claims expressed in this article are solely those of the authors and do not necessarily represent those of their affiliated organizations, or those of the publisher, the editors and the reviewers. Any product that may be evaluated in this article, or claim that may be made by its manufacturer, is not guaranteed or endorsed by the publisher.

## Abbreviations

NICU: neonatal intensive care unit
VUS: variant of unknown significance
SLNE: self-limited neonatal epilepsy
DEE: developmental and epileptic encephalopathy
CBZ: carbamazepine
IVS: intervening sequence or intron.
